# Supplementary material for: Recombinant Oxidase from Armillaria tabescens as a Potential Tool for Aflatoxin B1 Degradation in Contaminated Cereal Grain
Source: Toxins (Basel). 2023 Nov 30;15(12):678. doi: 10.3390/toxins15120678 (PMC10747862; doi:10.3390/toxins15120678)
Supplement: Supplementary file 1 [file toxins-15-00678-s001.zip › toxins-2725608-supplementary.pdf]

Supplementary Materials: Recombinant oxidase from *Armillaria tabescens* as a potential tool for aflatoxin B1 degradation in contaminated cereal grain

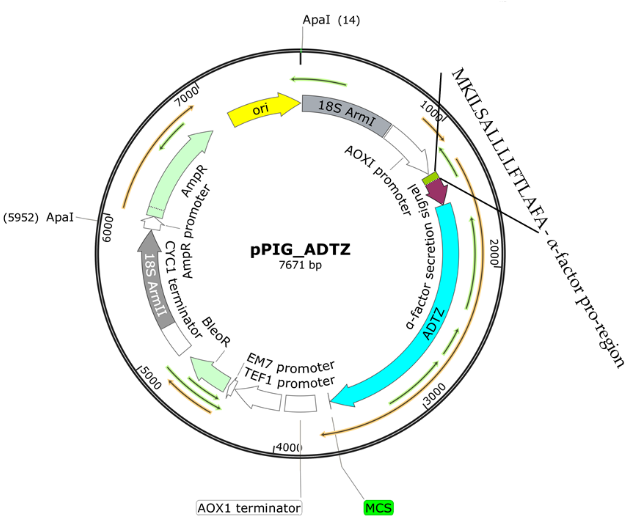

Figure S1. Map of the pPIGa-1-AFO plasmid

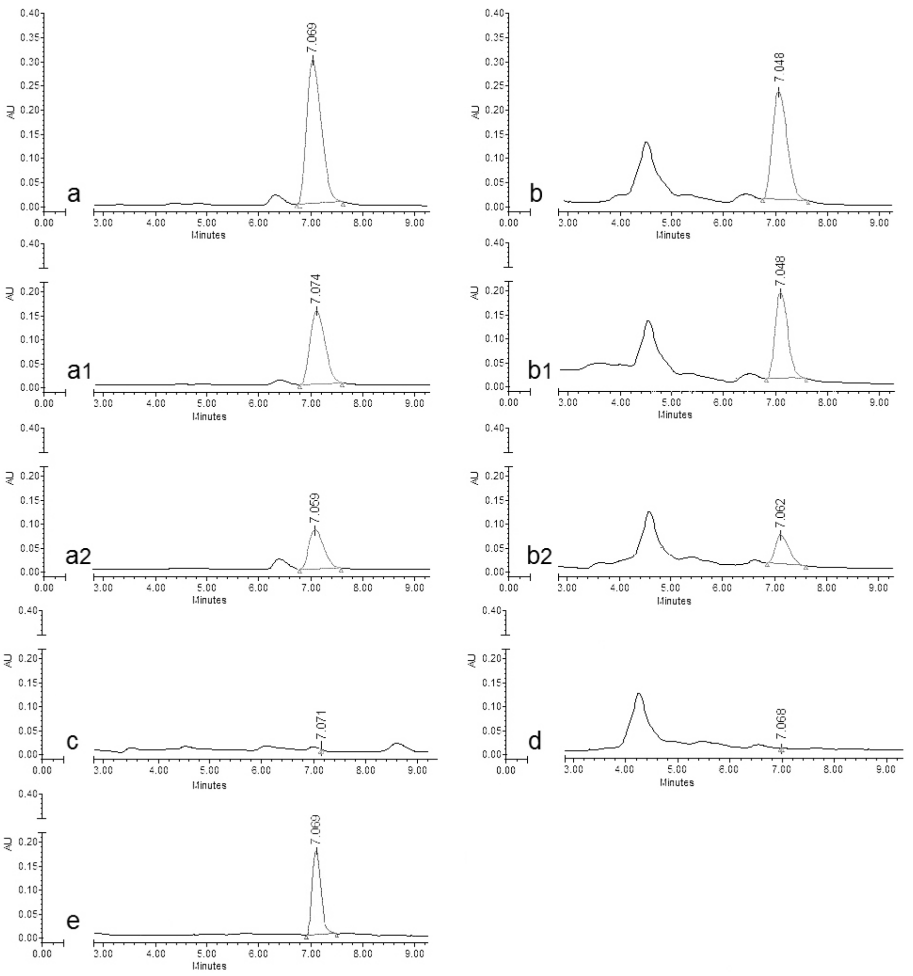

Figure S2. Chromatograms illustrating reduction of AFB1 content in extracts of wheat (**a1, a2**) and corn (**b1, b2**) grain inoculated with a toxigenic *A. flavus* (**a, b**) resulted from 24- (**a1, b1**) and 48-hour (**a2, b2**) grain exposure to recombinant AFO at pH 6.0 and 30°C. Chromatograms (**c**) and (**d**) show results of HPLC-analyses of extracts non-inoculated wheat and corn grain, respectively, as well as commercial AFB1 standard (**e**) under the same conditions.
